# Young men’s attitudes and behaviour in relation to mental health and technology: implications for the development of online mental health services

**DOI:** 10.1186/1471-244X-13-119

**Published:** 2013-04-20

**Authors:** Louise A Ellis, Philippa Collin, Patrick J Hurley, Tracey A Davenport, Jane M Burns, Ian B Hickie

**Affiliations:** 1 Brain & Mind Research Institute, The University of Sydney, 94 Mallett Street, Camperdown, NSW 2050, Australia; 2 Institute for Culture and Society, University of Western Sydney, Bankstown Campus, NSW 2751, Australia; 3 Young and Well Cooporative Research Centre, Abbotsford, VIC 3067, Australia; 4 Inspire Foundation, Camperdown, NSW 2050, Australia; 5 Orygen Youth Health Research Centre, Centre for Youth Mental Health, University of Melbourne, Parkville, VIC 3052, Australia

**Keywords:** Young men, Mental health, Help-seeking, Technology, Internet

## Abstract

**Background:**

This mixed-methods study was designed to explore young Australian men’s attitudes and behaviour in relation to mental health and technology use to inform the development of online mental health services for young men.

**Methods:**

National online survey of 486 males (aged 16 to 24) and 17 focus groups involving 118 males (aged 16 to 24).

**Results:**

Young men are heavy users of technology, particularly when it comes to entertainment and connecting with friends, but they are also using technology for finding information and support. The focus group data suggested that young men would be less likely to seek professional help for themselves, citing a preference for self-help and action-oriented strategies instead. Most survey participants reported that they have sought help for a problem online and were satisfied with the help they received. Focus group participants identified potential strategies for how technology could be used to overcome the barriers to help-seeking for young men.

**Conclusions:**

The key challenge for online mental health services is to design interventions specifically for young men that are action-based, focus on shifting behaviour and stigma, and are not simply about increasing mental health knowledge. Furthermore, such interventions should be user-driven, informed by young men’s views and everyday technology practices, and leverage the influence of peers.

## Background

Young men in Australia have poorer mental health than their female counterparts including higher rates of completed suicide, antisocial behaviour, and alcohol and substance misuse problems [1]. Although gender differences in help-seeking vary according to type of problem and source of help [2], young men are also less likely to seek help during adolescence and young adulthood: only 13% of young men aged 16 to 24 years seek help when experiencing mental health difficulty compared with 31% of young females [3]. The factors associated with poorer help-seeking practices in young men are complex [4]. Young men have poorer mental health knowledge and higher mental health stigma than young females [5,6]. Research also suggests that young men find it difficult to seek help as a result of culturally dominant (or hegemonic) masculine traits which place an emphasis on men to be independent, to suppress emotion, and show a lack of vulnerability [7-9]. For example, to be seen to endure pain and to be strong and resilient about mental health or emotional problems has been identified as a key practice of masculinity [9]. Furthermore, while these constructions of masculinity remain relevant, and are a backdrop for men’s illness behaviours, it should not mean that we adopt a view which positions men as victims of their own behaviour [10]. Rather than inherently blaming and therefore attempting to ‘re-educate’ young men, it is now being argued that greater focus should be placed on providing health services that are relevant and meet their needs [10-12].

### The Internet as a tool for health service delivery

The Internet has become an important tool for young people seeking health information [13,14]. Young people in Australia report they are twice as likely to seek help from the Internet than a professional [15]. The Internet has significant advantages as a method of interacting with young people: it can reach a wide audience; be accessed 24 hours a day at little or no cost; websites can be updated frequently; it is interactive; and, can link to other relevant resources [11,16]. Furthermore, the Internet may address the strong desire for independence and autonomy in males and provide a non-confrontational medium through which to seek help [17,18]. A recent review of online intervention programs for children and young people found that overall they had beneficial effects on their health behaviour; though these programs were generally focussed on improving physical health outcomes as opposed to mental health outcomes [19]. Two currently available online programs aimed at improving mental health outcomes, MoodGYM (http://www.moodgym.com.au; an interactive cognitive behavioural therapy program designed to prevent and decrease symptoms of depression and anxiety), and Reach Out Central (ROC; http://www.reachoutcentral.com.au; an interactive game utilising cognitive behavioural principles to develop practical coping skills for dealing with life stressors and improve mental health), have shown promise in trials with young people [11,20,21]. However, these studies have highlighted several important challenges for MoodGYM, ROC and other self-directed Internet programs; including how to ensure enough of the program is received and that users remain engaged with the program, as well as how to enhance the sustainability of any benefits. Immediate attention needs to be directed to improving usage and adherence rates [21], and new methods need to be explored which cater for young men’s mental health needs and expectations [22].

More needs to be known specifically about young men’s attitudes towards mental health and help-seeking and their use of technology if we are to create online interventions that attract and engage young men and enhance the sustainability of any benefits. Thus, the aim of the current study was to increase our understanding of young men’s attitudes and behaviours towards mental health, online habits and technology use, as well as their experiences of using the Internet for information, help or support, so as to inform the development of online mental health services for young men. The study addresses two key gaps in the existing literature: (1) empirical evidence of young men’s technology preferences and their attitudes and experiences towards online help-seeking for mental health problems; and, (2) rich qualitative data exploring a wide range of young male views and experiences in relation to mental health and help-seeking. Existing studies are typically quantitative and measure uptake and engagement, or involve randomised control trials of existing online interventions [23], and there is little data that focuses specifically on young men. A combination of quantitative and qualitative methods was therefore used allowing the study to examine both the breadth and depth of young men’s attitudes and experiences.

## Methods

### Online survey design and sample

A survey was administered online for a three-month period. Recruitment was achieved via online snow-ball sampling, leveraging young people’s high levels of Internet usage and existing social networks. Online sampling was used as a way of reaching young people who are normally difficult to access via random-digit dialling or panel methods, and as a way of reducing social desirability effects [24]. An advertisement was placed on Facebook, a popular online social networking site, and participants were encouraged to promote the survey to their peers, who then completed the survey and further promoted the study through their networks. The Facebook advertisement was specifically targeted to appear on the pages of Australian Facebook users between the ages of 16 to 24. The survey was also specifically advertised through youth serving organisations, including youth centres and clinics, online service providers, charities, colleges, universities and relevant government organisations, via a flyer and link to the survey which was distributed via email. Participants gave consent online and understood that their participation was voluntary, confidential and non-identifiable. This study received ethics approval from The University of Sydney Human Research Ethics Committee (Ref No. 11209).

### Survey measures

#### Interests and technology use

Participants were asked about their use of a range of technologies (eg. computers, playstation, Facebook) over the past three months (‘yes/no’).

#### Attitudes and behaviours in relation to mental health

Two sets of questions were selected from a recent national survey on mental health in Australia [25]. The first set of questions asked respondents what they would do if they thought a friend might be experiencing a mental health problem. Respondents were asked how likely it would be that they would suggest to their friend that they seek help from particular sources (eg. ‘family’, ‘friends’, ‘websites’, “doctor”), with items being rated on a five-point Likert scale (1 = ‘very likely’ to 5 = ‘very unlikely’). The second set of questions asked respondents whether they have ever talked about their problems on the Internet (‘yes/no’), and if so, whether chatting with other people via the Internet helped (‘yes/no’). Finally, respondents were asked how satisfied they were with the information/support they received on the Internet (1 = ‘very dissatisfied’ to 4 = ‘very satisfied’).

#### Technology and mental health

A set of questions was developed to measure preferences for receiving mental health information and support through technology. Respondents were asked ‘if you wee to access mental health information using technology, how would you want it presented?’ and the options included: ‘website with information and/or factsheets’; ‘website with a question and answer service that sends short message service (SMS) or emails’; ‘website with online clinic’; ‘interactive single player games teaching life skills’; ‘interactive multiplayer games teaching life skills’; ‘I don’t know’; ‘not a website’; and ‘other’.

#### Demographic variables

The survey also included a set of demographic questions relating to gender, age, location, ethnicity and employment status.

#### Focus group design and sample

A total of 17 focus groups were conducted involving 118 young men. Focus groups were advertised through a selection of youth serving organisations, including youth centres and clinics, schools, TAFEs, universities and businesses that hire young people, via a flyer. The sample was purposive in that it was deliberately designed to be diverse in terms of age (16 to 24 years), location (multiple states), ethnicity, and level of education (see Table 1).

**Table 1:** Location, profile and age ranges of 17 focus groups run nationally across Australia

**Location**	**Profile**	**Age range in years (mean)**
Sydney, NSW	Graduate employees at a leading accounting firm	22-24 (23.0)
Gosford, NSW	Local teenagers who occasionally use the youth centre	16-19 (17.4)
Lidcome, NSW	Call centre operators for a major alcohol company	21-24 (22.8)
Canberra, ACT	Trade apprentices	16-19 (17.9)
Sydney, NSW	Students attending s public high school	16 (16.0)
Sydney, NSW	Students attending a public high school	16-17 (16.8)
Wangara, WA	Trade apprentices from troubled backgrounds	16-21 (19.0)
Yangebup, WA	Regulars at a youth centre dedicated to developing leadership among Aboriginal young people	16-22 (17.9)
Maddington, WA	Local young people who frequent a youth centre in a low socio-economic area	16-19 (17.1)
Perth, WA	University students from private school backgrounds	19-20 (19.1)
Perth, WA	Young males identifying with diverse sexuality and gender.	17-24 (19.5)
Glen Forrest, WA	Students and apprentices	20-21 (20.1)
Perth WA	Students attending a Catholic high school	16-17 (16.2)
Broadmeadows. VIC	Recent Iraqi migrants	16-24 (20.7)
Torquay, VIC	Graphic designers for a major surfing brand	22-24 (22.7)
Box Hill, VIC	Young men who frequent a youth centre at Box Hill	18-24 (20.9)
Sydney, NSW	Lebanese Maronite Catholic Church group members	18-24 (20.1)

Each focus group involved four to 10 males and lasted 60 to 90 minutes. A schedule of questions developed in consultation with male Youth Ambassadors involved with Australia’s most accessed online youth mental health service (ReachOut.com) and was used for each focus group to explore the following themes: interests and technology use; knowledge of and attitudes towards help-seeking and mental health; as well as brainstorming innovative solutions to the problems identified. To address concerns regarding the willingness of participants to share their perspectives of sensitive topics in front of others, participants were asked to respond individually to questions about mental health by writing their answers down on a piece of paper. The focus groups were recorded, transcribed and analysed along with written notes.

### Data analysis

The survey data were analysed using the Statistical Package for the Social Sciences (SPSS 20.0 for Windows, Chicago, USA). Simple linear regression was used to investigate whether any significant age differences were present. Age was included as a continuous variable and a p-value of less than 0.05 was considered statistically significant. The focus group data was analysed thematically using complete transcripts of each session. Separately, two researchers with prior qualitative research experience systematically coded the transcripts applying brief verbal descriptions to small chuncks of data, and then identified themes which integrated substantial sets of these codings. The results were then compared and discussed until the generated themes were agreed upon [26]. This procedure was applied to ensure that the generated themes were identified and clustered in a way that was consistent with the views of more than one person and not simply a reflection of one researcher’s subjective interpretation.

## Results

### Quantitative results

#### Online sample

A total of 1,038 young people (aged 16 to 24 years) completed the survey (52.3% female; n=552; mean age=18.84 years; SD age=2.75). For the purposes of the current paper, only the data for young men was considered (n=486; age range=16-24 years; mean age=18.55; SD age=2.62). Participation for males varied across Australian States and Territories [41.0% were from New South Wales and Australian Capital Territory (n=198/483); 26.5% were from Victoria and Tasmania (n=128/483); 18.4% were from Queensland, (n=89/483); and 12.8% were from Western Australia, South Australia and the Northern Territory (n=68/483)]. Three percent of the male sample identified themselves as Aboriginal and/or Torres Strait Islander origin (2.9%; n=10/346); and, 22.3% spoke a language other than English at home (n=78/350). Sixty-six percent of the male sample were in full-time study at school, TAFE or university (65.6%; n=221/337), 13.6% were employed full-time (30 or more hours per week; n=46/337), and 8.9% were employed part-time (less than 30 hours per week; n=30/337).

#### Interests and technology use

The vast majority of males reported that they use mobile phones (96.3%), Ipod/mp3 players (88.5%), and computers (desktops, 80.1%; laptops, 81.6%) (see Table 2). More than half of the sample identified that they play video games (playstation, 53.0%; Nintendo/Wii, 49.9%; XBox, 51.0%). Age, however, was a significant predictor for game play with younger males being more likely to use a playstation [β=−.19 n=402, p<.001], XBox [β=−.21, n=402, p<.001], single player games {β=−.12, n=403, p=.016], multiplayer games [β=−.13, n=403, p=.011] and interactive games [β=−.18, n=403, p=.000] than older males (in these analyses a positive value of β indicates that there is a positive relationship between age and the dependent variable). The age differences are clearly shown when the results are broken down into narower age groups (see Table 2). Males reported that they are accessing video websites (85.1%) and information websites (79.4%) far more than forums (62.0%) and bulletin boards (39.2%). Facebook is by far the most popular social networking site (90.1%), with only 37.5% of the sample using MySpace and only 20.6% using Twitter. MSN is also still a popular way to communicate with other people (76.9%). Older males are more likely to access information websites [β=.25, n=403, p<.001] and use Twitter [β=.14, n=403, p=.006]; while younger males are more likely to use MySpace [β=−.15, n=403, p= 003] and MSN [β=−.35, n=403, p<.001].

**Table 2:** Technology use for males aged 16–24 years

	**n (%)**		
	**16-18 year olds**	**19-24 year olds**	**Total (16–24 year olds)**
Landline phone	211 (86.5)	110 (70.1)	321 (80.0)
Mobile phone	234 (95.4)	154 (97.5)	388 (96.3)
iPod/ Mp3 player	222 (90.6)	133 (85.3)	355 (88.5)
Playstation	146 (59.6)	67 (42.7)	213 (53.0)
Nintendo/Wii	131 (53.5)	70 (44.3)	201 (49.9)
Xbox	145 (59.2)	60 (38.2)	205 (51.0)
Desktop computer	204 (83.3)	119 (75.3)	323 (80.1)
Laptop computer	194 (79.5)	134 (84.8)	328 (81.6)
MSN	216 (88.2)	94 (59.5)	310 (76.9)
Skype	86 (35.1)	62 (39.2)	148 (36.7)
Twitter	42 (17.1)	41 (25.9)	83 (20.6)
Facebook	225 (91.8)	138 (87.3)	363 (90.1)
Myspace	107 (43.7)	44 (27.8)	151 (37.5)
Bebo	39 (15.9)	14 (8.9)	53 (13.2)
Single player games	174 (71.0)	94 (59.5)	268 (66.5)
Multiplayer games	149 (60.8)	71 (44.9)	220 (54.6)
Interactive games	168 (68.6)	81 (51.3)	249 (61.8)
Information websites	176 (71.8)	144 (91.1)	320 (79.4)
Video websites	206 (84.1)	137 (86.7)	343 (85.1)
Forums	153 (62.4)	97 (61.4)	250 (62.0)
Bulletin boards	98 (40.0)	60 (38.0)	158 (39.2)

Note. Row percentages used. Participants were able to select multiple options.

#### Attitudes and behaviours in relation to mental health

The most ‘likely’ or ‘very likely’ sources of help they would recommend would be: friends (86.6%); a counsellor (70.1%); doctor (67.1%); family member (63.5%); and websites (40.6%) (see Table 3). They would be least likely to recommend posters or pamphlets, a church leader, teacher, or community centre. Age, however, was a significant predictor for websites and church leader, with younger males being more likely to recommend websites than older males [β=−.15, n=397, p=.004], and older males being more likely to recommend a church leader than younger males [β=.11, n=396, p=.036].

**Table 3:** Sources of help young men aged 16–24 years would suggest to a friend with a mental health problem

	**n (%)**				
	**Very likely**	**Likely**	**Unlikely**	**Very unlikely**	**Don’t know**
Family	104 (24.8)	162 (38.7)	81 (19.3)	54 (12.9)	18 (4.3)
Friends	173 (41.6)	187 (45.0)	36 (8.7)	13 (3.1)	7 (1.7)
Websites	53 (12.9)	114 (27.7)	154 (37.5)	76 (18.5)	14 (3.4)
Telephone helplines	47 (11.2)	96 (23.0)	146 (34.9)	113 (27.0)	16 (3.8)
Posters or pamphlets	20 (4.8)	62 (14.9)	182 (43.8)	137 (32.9)	15 (3.6)
Teacher	32 (7.7)	73 (17.6)	175 (42.3)	114 (27.5)	20 (4.8)
Doctor	106 (25.2)	176 (41.9)	80 (19.0)	44 (10.5)	14 (3.3)
Community centre	33 (8.0)	72 (17.4)	173 (41.8)	116 (28.0)	20 (4.8)
Trusted community member	29 (6.9)	93 (22.2)	166 (39.7)	114 (27.3)	16 (3.8)
Church leader	38 (9.1)	52 (12.5)	112 (26.9)	194 (46.5)	21 (5.0)
Counsellor	115 (27.5)	178 (42.6)	63 (15.1)	48 (11.5)	14 (3.3)

Note. Percentages are given in parentheses. Due to some missing data, the total sample size for each source varied between 411 and 420.

Survey participants were asked whether they had ever sought help for their problems on the Internet. More than half of all male respondents reported that they had talked about their problems online (54.9%, n=212/386). Most said that talking online ‘helped’ (81.3%, n=169/208), and that they were ‘satisfied’ or ‘very satisfied’ with the online help they received (82.9%, n=174/210). Age was a significant predictor for seeking help online, with younger males being more likely to have talked about their problems online than older males [β=.16, t(384)=3.15, p=.002].

#### Technology and mental health

The survey also asked participants to indicate their preferences for receiving mental health information and support through technology. The top four responses were: website with information and/or fact sheets (48.1%, n=234); website with online clinic (38.5, n=187); website with information and multimedia content (29.6%, n=144); and website with question and answer service that sends sms or emails (28.8%, n=140). Older males were more likely to report that they wanted a website with information and/or fact sheets [β=.14, n=370, p=.007] and a website promoting physical wellbeing [β=.12, n=370, p=.027] than younger males.

### Qualitative results

#### Focus group sample

A total of 118 males (aged 16 to 24 years) from four Australian States [40.7% from Western Australia (48/118), 33.1% from New South Wales (39/118), 20.3% from Victoria (24/118), and 5.9% from Australian Capital Territory (7/118)] participated in the focus groups. Twenty-five% of participants had completed education or training beyond high-school (25.4%, 30/118), 61.8% were in some form of employment (73/118), with 33.9% percent working full-time (40/118), and 39.0% of participants identified with a cultural background other than Australian (46/118).

#### Interests and technology use

The vast majority of focus group participants indicated that they are enthusiastic and heavy users of technology. Across all focus groups, participants listed at least 10 different technology-based practices they regularly engage in. Most frequently reported was Internet use via computers, computer/console games, mobile phones and portable audio devices for a range of activities (eg. downloading movies and music, reading online news, blogs, watching sport and pornography, listening to podcasts and online shopping). Overall, few group differences were identified. However, older males tended to be more likely to report using technology for reading news, searching for restaurants, as well as for banking purposes than younger males.

Social networking and video sites were universally reported among focus group participants for socialising, pursing general interests and listening to music, although there was some diversity in the particular services being used. As reported by one young male:

*“Whenever I go on a computer, the first thing I open is Facebook and YouTube.”* (High school student)

Consistent with our quantitative survey results, the vast majority of focus group participants reported using Facebook, with only a small minority using Twitter and MySpace. As highlighted by one participant: “not MySpace; that’s so last year” (High school student). This comment is consistent with the assertion of Boyd that while particular social networking services will come and go, it is the activity of communication and socialising that is important to these young people [27]. Many participants also indicated that they are enthusiastic about searching “funny stuff” and following a trail of linked videos:

*“I just type in ‘lol’ or ‘funny’ and watch like 30 videos…it takes me on atangent.”* (High school student)

#### Beliefs about mental health

The focus groups explored young men’s beliefs about health and mental health with some very consistent themes emerging. Firstly, consistent with previous research [28], most focus group discussions of ‘what it means to be healthy’ were dominated by references to physical fitness and diet, revealing that young men generally have a narrow conception of health. However, more educated participants and students studying Personal Development, Health and Physical Education (PDHPE) in high school tended to have a more holistic, multidimensional concept of health. One high school student summed it up well:

*“It’s all about being spiritually, mentally, physically and socially healthy.”* (High school student)

Secondly, across all 17 focus groups, the term ‘mental health’ had overwhelmingly negative connotations among focus group members and was associated, with things like *“insanity”*, *“being crazy”*, *“straight jackets”*, *“mental institutions”* and *“unstable people”*. While most respondents acknowledged that mental health problems are relatively prevalent in the wider community, many of those tended to believe they would never be personally affected a mental health difficulty:

*“I can‘t really see it affecting me.”* (University student)

*“I can‘t really imagine having a mental health problem to be honest.”* (Call-centre operator)

In addition, when asked what they know about mental health, many participants said they knew relatively little:

*“Mental health? Not that much.”* (High school student)

*“I don’t even know what mental health means.”* (Youth Centre member)

*“I don’t know a lot about it.”* (University student)

Nevertheless, depression was correctly identified in all but two focus groups as a common mental health condition for young people [1]. Some participants also noted that depression is experienced mainly by young girls, and to a lesser extent older men. For example, one participant commented:

*“I think younger girls [get depressed] more, and older guys…I’ve seen heaps of girls when I was at school that cut their wrists and stuff. And that’s the first indication that they’ve got something wrong to me.”* (Trade apprentice)

#### Attitudes towards help-seeking

Focus group discussions provided insights into the gap between existing help options and young men’s actual help-seeking, which can be summarised in four key themes: (1) notions of masculinity; (2) communication barriers; (3) the role of self-help strategies; and, (4) perceptions of current mental health services.

Firstly, across all 17 focus groups, participants indicated that they would find it difficult to seek help as a result of culturally dominant masculine traits that place an emphasis on males to be *“strong”* and to *“not show any emotion”*, a finding that is consistent with previous research [7-9]. Participants’ comments highlighted that help-seeking is associated with *“weakness”* and a *“loss of manhood”*. For example:

*“…[to seek help is] almost an admission of weakness. You may not want to show that weakness to certain people, because that might change their opinion of you.”* (Graduate accountant)

*“The first time you to [to a counsellor] you think ‘I’m not going to be a man anymore’.”* (High school student)

One other young male summed it up well:

*“…[seeking help] just doesn’t fit the male stereotype.”* (Youth centre member)

Across a number of focus groups, there were participants who expressed strong views that they wouldn’t need – or seek – help under any circumstances:

*“For me, I just don‘t feel like that there would be any issue that I would need to go to someone externally for.”* (Graduate accountant)

Some also expressed a tendency for self-denial in relation to mental health issues:

*“…I guess there‘s still a stigma of mental health being a weakness and not something you want to show. I realise that it‘s not but it‘s just something that I‘d find difficult coming to terms with - like ‘that kind of shit doesn‘t happen to me’.”* (University student)

A second major theme that emerged related to communication barriers. Regardless of age, geographic location or level of education, many participants indicated that they would be uncomfortable *“talking”* about their problems with either their friends or a professional:

*“For some reason its harder for dudes to open up and express their feeling; maybe the way we communicate is different to girls; we communicate through sport and physical activity and stuff whereas girls will sit down and talk about their problems.”* (High school student)

Furthermore, participants’ comments revealed that “talking” is generally considered a feminine characteristic:

*“…[Talking about your problems] is not a thing that’s really accepted. Guys don’t want to feel like they’re all girly.”* (Trade apprentice)

As well as not wanting to discuss mental health issues themselves, participants across various focus groups indicated that they would not want to hear others talk about mental health issues, particularly if they were talking with someone other than a *“best mate”*. For example, disclosing personal or sensitive information to someone else who is not their closest friend was described in one focus group as *“over-sharing”* and strongly discouraged. Participants who held such views indicated they would be unreceptive listeners:

*“Even if one of my friends is just whinging about something, I’m like ‘Man, get over it, I don’t want to hear it.”* (Call centre operator)

Many other participants explained that they would prefer not to directly raise an issue with a friend showing signs of poor mental health. Rather, they would first attempt to ‘help’ their friend by encouraging them to participate in sport, socialising or drinking as opposed to engaging with the cause of the problem directly. However, participants across virtually all focus groups indicated that would address the issue directly or actively encourage their friend to seek professional help if they felt it was absolutely necessary. Interestingly, these discussions also revealed that some would only see a professional if a close friend or family member actively encouraged them to do so:

*“Well, I know that my friends and family would always be honest with me, so even if I couldn’t see it in myself they’d tell me that I need to see someone. I’d like to think that I’d accept that and take on board those thoughts of theirs. So it would probably take that for me to go.”* (Graphic designer)

This suggests a ‘catch-22’ situation whereby these young men would tend to resist encouraging a friend to seek help but at the same time would require a close friend’s intervention if they themselves were going through a tough time.

The third major theme that emerged related to the role of self-help strategies. The vast majority of focus group participants expressed the view that dealing with one’s own problems was preferable to seeking help from others:

*“People have different mentalities, mine is ‘I can resolve my issues myself’, so I don’t need to seek help.”* (Call centre operator)

*“I wouldn’t like to speak about my problems. I really like to do things my own way, independently.”* (High school student)

Finally, across all focus groups, participants displayed a range of negative attitudes in relation to mental health professionals; specifically, counsellors and psychologists. When asked to list the persons that participants would feel comfortable discussing personal problems with, very few mentioned counsellors; a finding that is consistent with previous research involving young people [29]. Participants generally expressed low trust and lack of confidence in professionals’ maintaining confidentiality and ability to actually help. Some participants said they would not want to pay for services and were sceptical of the professional’s motives (i.e. professionals have a monetary incentive to keep clients coming to sessions); others believed that they could get the same support for free from close friends, family or online. The logistical issues of seeking professional support were also sighted as a significant barrier. Having to make an appointment, travel to an unfamiliar location and then discuss emotional issues at a specific time were all reasons given for not accessing professional services. Mental health professionals were described as older, with different life experiences and hard to relate to. When asked what would make a professional more appealing participants felt they should be *“down to earth”* (eg. *“not use big, medical words”*), non-judgemental and have experienced a mental health problem themselves. These factors contribute to the view across all groups that professional support services are the ‘option of last resort’.

#### Using the Internet for information, help or support

Across all focus groups, participants indicated a willingness to seek information and support from the Internet:

*“I’d prefer to talk to someone on the Internet and then maybe make my way to a counsellor or a psychiatrist, rather than just jumping straight in the deep end and going to a psychiatrist.”* (Trade Apprentice)

In this way, the Internet was seen as a gateway to information and support. Some also highlighted the importance of peer rating and reviews of both online and offline help-options:

*“I don’t really have any info on who’s good, who’s not, what’s good, what’s not. I don’t really know anything about it. So I might do an Internet search to see if I can find anyone talking about going to see a counsellor or a psychiatrist.”* (Call centre operator)

In line with previous research, most participants indicated the need for online information and support services to be an anonymous process and fears of being identified when seeking help were key themes when the value of seeking information and support online was discussed [17].

Finally, focus group participants brainstormed strategies for using technology to address some of the barriers to help-seeking. These were grouped under common themes with three key insights emerging. Firstly, most participants indicated that they would be fearful of being judged by their peers, family or a professional but said this could be mitigated by being able to seek information and help anonymously online. Secondly, many participants emphasised the importance of interventions being relevant and relating to their everyday lives and interests. This could include delivering mental health content in young men’s online communities of interest (eg. sports sites, music sites or male magazines). Some also recommended male role models, such as boxers, sports players, and actors. Thirdly, many participants indicated a preference for action-based rather than talk-based strategies. One participant requested the following:

*“…really advanced search tool or questionnaire – or self-diagnosing thing… and it would link you to some kind of page with testimonials”* (Youth centre user).

Broadly, participants were interested in opportunities to build skills in ‘how’ to identify, discuss and manage mental health issues.

## Discussion

This study is unique in its focus on exploring and understanding young men’s attitudes and behaviours in relation to technology use and mental health. Methodologically, the use of both quantitative and qualitative findings is a strength of this study.

Not surprisingly, this study confirms that technology is an integral part of young men’s lives. The survey data is consistent with national data on technology access and use [30,31], and the focus groups provide insight into the complex ways in which technology is integrated into their everyday lives as well as the ways in which they use technology to mediate the different issues and experiences they face. Both the survey and focus group data were consistent in demonstrating that young men use technology predominately for entertainment and connecting with others. Facebook and YouTube appear to be a key source of entertainment and an integral part of their lives. However, it is important to recognise that the popularity of particular sites can change quickly, as the continued demise of MySpace demonstrates, and emphasises the importance for research in this area to be undertaken regularly to keep up with the ever-changing landscape [32].

The findings of this research also build on existing studies [2,6,33-35], particularly in terms of the role of the Internet for promoting help seeking in young men. Firstly, consistent previous research [36], the survey data indicated that if young men were to refer help to someone with a mental health problem, they would most likely recommend an informal source of help (ie. a friend) rather than a physician or mental health professional. Likewise, participants in the focus groups displayed strong resistance and lack of skills to seek mental health information, support and help for themselves when needed [5]. They associate mental health with illness and pathology and as something that happens to ‘other people’. The focus group data suggested that they would be unlikely to seek professional help for themselves, citing a preference instead for self-help and action-oriented strategies; though they may be more likely to seek professional help if a friend intervened and actively encouraged them to do so. Although the findings were remarkably consistent across all 17 focus groups, there was evidence to suggest that those with a higher level of education or those currently studying PDHPE may have more informed understandings of mental health. Thus, these findings correspond with recommendations made by Rickwood et al. [29], and point to the need for interventions to reach young men beyond formal educational settings, focus on behaviour change through self-help and action-oriented strategies, and leverage the significant role that peers play in the pathway to professional services.

Across all focus groups participants presented negative views on professional services, and a related perception that seeking professional help challenges their sense of masculinity. As identified in previous research, a variety of masculinity ideologies, norms and gender roles appear to play a part in discouraging males from seeking professional help [37]. However, young men’s fears of being judged as weak or ‘unmanly’ could also be the key to building knowledge and skills that support help-seeking. Research participants themselves suggested that interventions should not be explicitly branded as ‘mental health interventions’, but rather tap into male sub-cultures and focus on building strength or improving performance. Interventions must therefore be relevant and engaging for young men and should carefully balance peer-recommendation with anonymity. Furthermore, interventions which are action-based, rather than talk-based may be more engaging.

In addition, the survey and focus group data was consistent in indicating a strong willingness for young men to use the Internet to find mental health information and support. The Internet addresses their desire for anonymity and self-help. Notably, the survey data suggested that when young men obtain information and help-seeking online, they are satisfied with the help they receive, which suggests that the Internet is an appropriate setting to engage with young men around their mental health.

Finally, the findings of this study point to some important insights that can be used to inform strategies to use the Internet to promote mental health and help-seeking among young men. Internet supported strategies to support mental health have increased tremendously over the past decade and now offer a real alternative, or supplement, to traditional, face-to-face therapeutic interventions. Existing interventions take a number of different forms and can be broadly distinguished as: primarily self-guided web-based interventions (eg., mental health information websites and online treatment programs without therapist interaction); online counseling (via textual communication between a therapist and consumer); or Internet-operated therapeutic software that uses advanced computer capabilities (eg., for robotic simulation of therapists providing dialog-based therapy, gaming, and three-dimensional virtual environments) [38]. The findings from this study suggest that we may need to look at gaming and three-dimensional virtual environments as inspiration to enhance enjoyment for and engagement with young men. The term “gamification” has been adopted as an umbrella term for the use of gaming elements in non-gaming systems to improve user experience and engagement. However, Monk et al. [39] introduced a note of caution stating that the challenge for research in this field is to “systematically address hedonic (non-utalitarian) requirements and combine them with goal oriented requirements”. This has particular relevance in a mental health context where designers must place an emphasis on engagement with the treatment, rather than engagement with the technology [40]. Our study is concordant with current views that Internet interventions may be more likely to be successful for young men if they provide: a more interactive experience (with richer and more varied experiences, immediate graphical feedback, and the ability to engage actively rather than passively receiving content); a more personal experience (including content that is tailored to the user’s needs, and gives users a sense of control and ownership, and allowing them to choose their own pathway through the intervention); and facilitate contact with some kind of community (most obviously peers who are suffering, or have previously suffered from, similar difficulties [40]. However, further research is needed to build on these findings.

Three potential limitations should be considered when interpreting the results of the study that relate to the sampling methodology. First, given that we recruited participants for the survey using online methods, our sample was limited to young people with Internet access. Nevertheless, Internet access and use in Australia is very high: 97% of young people have personal access to the Internet [30] and the results for the survey concerning ICT use are comparable with previous research [31,41]. Secondly, our survey sample was recruited via Facebook advertising and snowball sampling which again raises questions about the generalizability of the results. A third potential limitation relates to the representativeness of our focus group sample. An analysis of the Australian Bureau of Statistics SEIFA Index [42] in relation to participants’ home address postcodes suggests that our sample may have been skewed towards young men from higher socioeconomic backgrounds. However, postcode measures of socioeconomic status are notoriously problematic, but due to ethical concerns, we were unable to collect more accurate measures of socioeconomic status, such as family income. However, the primary aim of the qualitative component of this study was not to recruit a statistically representative sample of all young men across Australia, but rather to include the perspective of a broad range of young men in order to capture the richness and complexity of young men’s attitudes and behaviours in relation to technology use and mental health, and to use these findings to build on the survey results.

## Conclusions

This study suggests that there may be powerful views towards mental health and help seeking that are gender specific. Though further work must now be undertaken, this study suggests that there may be a compelling need for gender-specific strategies and interventions. These should be informed by young men’s views and technology practices and take into account the important role that peers play in the help-seeking process. Previous research from Australia indicates an increase in awareness of mental health issues, particularly for those who have been the subject of extensive public health campaigns, such as depression [43]. However, the results of this study clearly indicate that although young men may have better awareness and understanding, the real challenge is to design interventions that are action-based, seen as relevant, and focus on shifting behaviour and stigma. In conclusion, the findings of this study point to some important insights that can be used to inform strategies to use the Internet to promote mental health and help-seeking among young men.

## Competing interests

The authors declare that they have no conflicts of interest.

## Authors’ contributions

PH carried out the interviews and participated in the qualitative data analysis. PC led the qualitative data analysis and LE led the quantitative data analysis and wrote the first draft of the article. All authors participated in the drafting of the final article. All authors read and approved the final manuscript.

## Pre-publication history

The pre-publication history for this paper can be accessed here:

http://www.biomedcentral.com/1471-244X/13/119/prepub
